# Long-term outcomes of internet-delivered cognitive behaviour therapy for paediatric anxiety disorders: towards a stepped care model of health care delivery

**DOI:** 10.1007/s00787-020-01645-x

**Published:** 2020-09-22

**Authors:** Maral Jolstedt, Sarah Vigerland, David Mataix-Cols, Brjánn Ljótsson, Tove Wahlund, Martina Nord, Jens Högström, Lars-Göran Öst, Eva Serlachius

**Affiliations:** 1 Child and Adolescent Psychiatry Research Centre, Department of Clinical Neuroscience, Karolinska Institutet, Gävlegatan 22, 113 30 Stockholm, Sweden; 2 Stockholm Health Care Services, Stockholm County Council, Stockholm, Sweden; 3 Division of Psychology, Department of Clinical Neuroscience, Karolinska Institutet, Stockholm, Sweden; 4 Department of Psychology, Stockholm University, Stockholm, Sweden

**Keywords:** Child psychiatry, Anxiety disorders, Cognitive behaviour therapy, Health services accessibility, Telemedicine

## Abstract

Internet-delivered cognitive behaviour therapy (ICBT) is emerging as a powerful tool to fill the gap between demand and availability of evidence-based treatment for paediatric anxiety disorders. However, it is still unclear how to best implement it in routine clinical care. 123 children (8–12 years) with anxiety disorders underwent a 12-week ICBT programme with limited therapist support. Participants were assessed 3- and 12-month post-ICBT (3MFU and 12MFU, respectively). Non-remitters who still fulfilled diagnostic criteria for their principal anxiety disorder at 3MFU were offered additional manualised “face-to-face” (F2F) CBT. The aim of the study was to emulate a stepped-care model of health care delivery, where the long-term treatment gains of ICBT as well as the potential benefit of proving addition treatment to non-remitters of ICBT were evaluated. Remitters of ICBT (*n* = 73) continued to improve throughout the study period (pre-ICBT to 12MFU; Cohen’s *d* = 2.42). At 12MFU, 89% (*n* = 65) were free from their principal anxiety disorder. Of all the participants classed as non-remitters at 3MFU (*n* = 37), 48.6% (*n* = 18) accepted the offer to receive additional F2F CBT. These participants also improved with a large effect from pre-ICBT to 12MFU (Cohen’s *d* = 2.27), with the largest effect occurring during F2F CBT. At 12MFU, 83% (*n* = 15) were free from their principal anxiety disorders. The majority of non-remitters declining additional F2F CBT (63.2%; *n* = 12) did so due to already receiving treatment at their local CAMHS, prior to 3MFU. The effects of ICBT for anxiety disorders are durable at least up to 1 year after the end of treatment. Patients who fail to fully benefit from ICBT improved further with additional F2F sessions at our clinic, suggesting that it may be feasible to implement ICBT within a stepped-care model of health care delivery.

**Electronic supplementary material:**

The online version of this article (10.1007/s00787-020-01645-x) contains supplementary material, which is available to authorized users.

## Introduction

Paediatric anxiety disorders are common [1] and often accompanied by a range of related problems if left untreated [2]. Development of early, accessible and cost-efficient interventions is therefore imperative. Cognitive behaviour therapy (CBT) is an efficacious first line treatment for anxiety disorders in children [3] but access to it is limited [4]. Remote delivery of CBT via the Internet (ICBT) is both efficacious and cost-effective for young people with anxiety disorders [5, 6] and has the potential to greatly increase the availability of treatment [7]. However, the long-term effects of ICBT for paediatric anxiety disorders have rarely been studied. To date, only three trials have investigated whether the treatment gains of ICBT for paediatric anxiety disorders were maintained at long-term follow-up [8–10]. All three studies suggested that treatment gains were not only maintained but there were indications of further improvement at follow-up. However, these studies had substantial data loss at follow-up and none recorded additional treatments received during the follow-up period, casting some doubt about the long-term effects of ICBT.

Assuming that the effects of ICBT for anxiety disorders are durable, it is still unclear how to best implement this novel treatment modality in routine clinical care. ICBT is often quoted as a potential early intervention in the context of a stepped-care model [11], where patients are first offered a low-intensity intervention, reserving higher intensity treatments to more complex cases or to those who fail to benefit sufficiently [12]. Though stepped-care approaches may intuitively seem like an ideal model for psychiatric service delivery, evidence for their feasibility in real-world settings is scarce [12–15].

This study reports the naturalistic 1-year follow-up data from participants in a large randomised controlled trial (RCT) of ICBT for paediatric anxiety disorders [6]. As part of the study protocol, all participants (*N* = 131) received ICBT either immediately post-randomisation or after crossover from the control intervention. Emulating a stepped-care mode of healthcare delivery, participants who required additional treatment at 3-months follow-up (3MFU), were systematically offered face-to-face CBT (F2F CBT) at our clinic. Additional treatments were carefully recorded throughout the follow-up period for all patients. Our specific research questions were: (1) For patients who were in remission at 3MFU, were their treatment gains maintained up to 12 months after treatment completion? (2) For patients classed as non-remitters at 3MFU, was additional F2F CBT associated with improvements in symptoms and functioning?

## Methodology

### Participants

Participants with a principal diagnosis of separation anxiety disorder, generalised anxiety disorder, specific phobia, social anxiety disorder or panic disorder were recruited through newspaper advertisements and referrals from the child- and adolescent mental health service (CAMHS) or primary care centres in Sweden. The study inclusion- and exclusion criteria are listed in Supplementary Table 1. Caregivers provided written consent and children provided verbal assent prior to participating in the study. The study protocol was approved by the Stockholm Regional Ethical Review Board (reference numbers 2014/1885-31 and 2015/316-31/1) and the trial was registered at ClinicalTrials.gov (number NCT02350257).

### Study design

For full details of the study design, please see [6]. Briefly, participants were randomly allocated to receive either 12 weeks of ICBT (*n* = 66) or of an active control condition called Internet-delivered child directed play (*n* = 65). Participants allocated to the control condition were offered to cross over to ICBT immediately after the primary endpoint (week 12), regardless of whether they were in remission. Fifty-seven participants, of whom six were classed as being in remission, accepted the offer and started a course of ICBT. The remaining eight dropped-out from the study during or directly after the control condition and did not provide further data. In total, a pooled sample of *N* = 123 participants received ICBT (i.e., 66 immediately and 57 crossing over). Of these 123 participants, 117 still met diagnostic criteria for their principal anxiety disorder at the time they started ICBT (Supplementary Fig. 1).

**Fig. 1:**
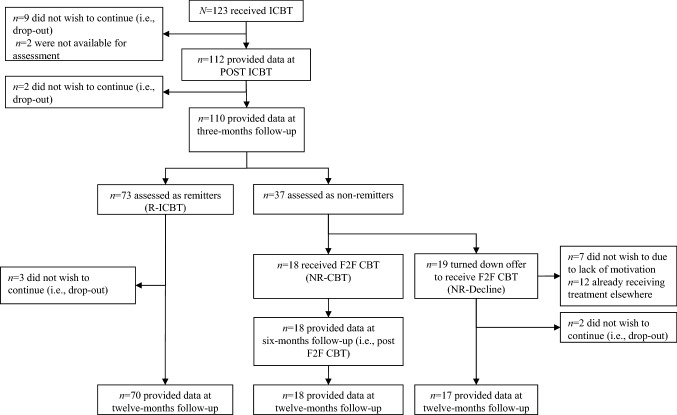
Participant flow through-out the trial. *ICBT* Internet-delivered Cognitive Behaviour Therapy; *F2F CBT* Face-To-Face Cognitive Behaviour Therapy; *R-ICBT* Remitters of Internet-delivered Cognitive Behaviour Therapy; *NR-CBT* Non-remitters of Internet-delivered Cognitive Behaviour Therapy receiving additional face-to-face Cognitive Behaviour Therapy; *NR-Decline* Non-remitters of Internet-delivered Cognitive Behaviour Therapy declining the offer to receive additional face-to-face Cognitive Behaviour Therapy. Participants already receiving treatment elsewhere started treatment prior to 3-months follow-up assessment

Participants were recruited to the original RCT during March 11, 2015–October 21, 2016. The period of follow-up lasted from 1 October, 2015 to April 2, 2018; see Fig. 1 for a flow chart and assessment points throughout the present trial. Participants still meeting diagnostic criteria for their principal anxiety disorder at 3MFU were offered to receive additional F2F treatment at our clinic.

### Measures

The primary outcome measure was the Clinician Severity Rating (CSR) of the principal anxiety disorder derived from the Anxiety Disorder Interview Schedule for DSM-IV: parent and child versions (ADIS-C/P) [16]. In ADIS, the severity of each diagnosis is rated with the CSR on a 9-point Likert scale, ranging from 0 (“Absence of symptoms/No disturbance in functioning/No disability”) to 8 (“Very severe symptoms/Very severe disturbance in functioning/Very severely disabling”). A rating of four or higher indicates that the child meets diagnostic criteria for the disorder. The full range (0–8) was assigned in the current trial. The ADIS C/P has shown good to excellent test–retest reliability and inter-rater reliability [17] as well as concurrent validity [18]. The inter-rater reliability in this trial based on the principal anxiety disorder was good (ICC = 0.77; 95% CI = 0.49–0.89) for CSR and fair (*κ* = 0.57, 95% CI 0.38–0.76) for assessing diagnostic status.

Secondary outcome included diagnostic status assessed with the ADIS; measures of clinician assessed functional impairment with *the Children’s Global Assessment Scale* (CGAS) [19]; self and parent reported anxiety symptoms with *the Revised Children’s Anxiety and Depression Scale* (RCADS-C/P) [20, 21]; self and parent reported functional impairment with *the Work and Social Adjustment Scale* (WSAS-C/P) [22]; as well as self and parent reported quality of life with the *KIDSCREEN-10* [23]. The RCADS-C/P consists of two subscales, the major depression subscale and the total anxiety subscale. The latter was used in the current trial. Parental anxiety and depression were measured with *the Hospital Anxiety and Depression Scale* (HADS) [24]. We also carefully documented whether the child had received *additional treatment* elsewhere, defined as either CBT or medication with SSRI including change in dosage during the follow-up period; and whether any adverse events were experienced during ICBT or F2F CBT; see Supplementary Table 2 and 3 for an overview of the measures used and when during the study period they were administered.

### Interventions

#### Internet-delivered CBT

The ICBT protocol (BiP Anxiety) is a completely web-based, therapist-guided self-help CBT programme for children with anxiety disorders and their caregivers. The treatment contains 12 consecutive modules for the child to complete together with one or both parents and 12 separate modules directed to the parent only. The duration of the treatment is 12 weeks and one module needs to be completed before gaining access to the next. The intervention primarily consists of self-guided exposure therapy with asynchronous and personalised therapist support provided on a weekly basis, via messages in the treatment platform and worksheets within the programme. For a summary of the content of the BiP Anxiety programme; see Supplementary Table 4.

#### Face-to-face CBT for non-remitters

According to the study protocol, all participants classed as non-remitters (still meeting diagnostic criteria for their principal anxiety disorder) at the 3MFU were offered additional manualised F2F CBT at the Stockholm Child and Adolescent Psychiatry Research Centre, a specialised research unit within CAMHS in Stockholm. The F2F CBT manual had the same treatment content as the ICBT programme, with emphasis on exposure, but was delivered in a traditional face-to-face format. Therapists used a structured manual with instructions as well as worksheets that could be used throughout the treatment. Participants were offered up to 10 sessions of F2F CBT (60 min/session) during 12 weeks and treatment included both the child and at least one parent. For all participants, the first session started with a joint discussion about the possible reasons why ICBT had not had the desired effect, followed by collaborative treatment planning (e.g., which components to include, how many sessions at each phase, type and amount of parental involvement). The last session mainly focused on maintenance of treatment gains and relapse prevention strategies. Disorder specific components, such as imaginal exposure for generalised anxiety disorder, social skills training for social anxiety disorder and interoceptive exposure for panic disorder, as well as cognitive techniques could be administered when needed. For details on specific treatment components that were delivered; see Supplementary Table 5.

### Analysis

#### Research question 1

For participants in remission at 3MFU (R-ICBT; *n* = 73), a piecewise linear mixed model (LMM) [25] was fitted to determine within-group symptom change over time on the different continuous variables. LMM models use all available data and are ideally suited to handle missing data. The model included two knots to detect and specify the change that occurred between different time points, specifically from (1) pre- to post-ICBT, (2) post-ICBT to 3MFU and (3) 3MFU–12MFU. We also ran an additional LMM including the receipt of any additional treatments during the follow-up period (coded yes/no) as a covariate.

#### Research question 2

Because not all patients classed as non-remitters accepted the offer of additional F2F CBT post hoc analyses were conducted on this sub-sample. Two separate LMM models were used. For those who received F2F CBT (NR-CBT; *n* = 18), the LMM included an additional knot/spline (i.e., total of three knots) to specify the change from 3 to 6MFU (i.e., from pre- to post-F2F CBT). For participants declining the offer to receive additional CBT, despite still fulfilling criteria for their principal anxiety disorder (NR-Decline; *n* = 19), the LMM was fitted with only two knots.

#### Linear mixed models

In an additional post hoc analysis, three separate LMM were fitted without any knots, to determine the total change from pre-treatment to 12MFU for each of the three groups (R-ICBT, NR-CBT and NR-Decline). Effect sizes (Cohen’s *d*) for all changes, between the different time points, were calculated with the estimates derived from the LMM together with the pooled observed standard deviation [26]. When available, measures reported during treatment were included in all the conducted LMM. Further, all LMM were built by starting with a fixed intercept and fixed effect of time and then sequentially adding (1) a random intercept and/or (2) a random effect of time. The final model for each fitted LMM was determined by using the chi-square goodness-of-fit test.

#### Other considerations

In this study, remission was defined as no longer fulfilling diagnostic criteria at a given assessment point (i.e., CSR rating < 4) and is presented for both principal anxiety disorder as well as all anxiety disorders. Univariate ANOVA with post hoc Bonferroni multiple comparisons (continuous measures) and Kruskal–Wallis test with post hoc Mann–Whitney *U* test with Bonferroni adjusted alpha value (categorical measures) were used when comparing three groups (R-ICBT, NR-CBT and NR-Decline). Participants with the missing data were compared with those who did provide data at 12MFU (yes/no) by conducting independent samples *t* tests on the whole sample (*N* = 123). Assumptions for parametric tests were tested by using normal probability plots, residual plots and Cook’s distance. Statistical significance was set at *p* < 0.05 and 95% CI was used. All statistical analyses were conducted in SPSS version 25.

## Results

### Clinical characteristics

The demographic and clinical characteristics of the total sample and the various subgroups according to remission status are summarised in Table 1. Pre-treatment comparisons showed that participants in the NR-Decline group included more children with social anxiety disorder as their principal diagnosis, when compared with participants in the R-ICBT group (*z* = 2.58; *p* = 0.010). Participants in the NR-Decline group also had higher clinician severity rating (CSR) of the principal anxiety disorder as compared to participants in R-ICBT (mean difference = 0.59; *p* = 0.018) and a higher number of anxiety diagnoses compared to both participants in R-ICBT (mean difference = 0.96; *p* = 0.002) and NR-CBT (mean difference = 0.91; *p* = 0.030). No other significant differences at pre-treatment were observed. Severity of the anxiety disorder (*t* = 0.15, *p* = 0.882), degree of functional impairment (*t* = 1.00, *p* = 0.320) and number of comorbid diagnoses (*t* = 1.38, *p* = 0.171) did not differ significantly when comparing those with the missing data at 12MFU as compared to those who provided data.

**Table 1:** Demographic Features and Clinical Characteristics at pre-treatment

	Total sample *N* = 123	R-ICBT *n* = 73	NR-CBT *n* = 18	NR-Decline *n* = 19	*F* value	Kruskal–Wallis	*p* value
Female, *n* (%)	65 (52.8)	37 (50.7)	13 (72.2)	9 (47.4)		3.03	0.220
Age, *M* (*SD*)	9.93 (1.35)	9.89 (1.37)	9.94 (1.47)	9.89 (1.20)	0.01		0.998
Previous contact with CAMHS, *n* (%)	61 (49.6)	33 (45.2)	9 (50.0)	11 (57.9)		0.99	0.609
Ongoing medication, *n* (%)	12 (9.8)	7 (9.6)	1 (5.6)	3 (15.8)		1.11	0.575
Principal anxiety disorder, *n* (%)							
SEP	48 (39.0)	34 (46.6)	5 (27.8)	4 (21.1)		5.23	0.073
SAD	25 (20.3)	11 (15.1)	5 (27.8)	8 (42.1)		6.85	0.033
GAD	23 (18.7)	11 (15.1)	3 (16.7)	5 (26.3)		1.33	0.515
SP	20 (16.3)	12 (16.4)	4 (22.2)	2 (10.5)		0.92	0.632
PD	7 (5.7)	5 (6.8)	1 (5.6)	0 (0)		1.36	0.507
Number of diagnoses, *M* (*SD*)	1.81 (1.18)	1.60 (0.83)	1.67 (0.69)	2.58 (1.84)	6.38		0.002
CSR score, principal anxiety disorder, *M* (*SD*)	4.56 (0.81)	4.41 (0.85)	4.72 (0.83)	5.00 (0.67)	4.33		0.016
Depressive symptoms, *M* (*SD*)	6.85 (4.22)	6.46 (4.08)	6.33 (5.02)	7.83 (4.36)	0.95		0.391
Parental anxiety and depression, *M* (*SD*)	8.87 (6.84)	8.60 (6.22)	8.28 (7.43)	8.74 (6.66)	0.03		0.975
Educational level, parent, *n* (%)							
< 12 years	7 (5.7)	4 (5.5)	0 (0)	1 (5.3)		1.02	0.601
12 years	16 (13.0)	9 (12.3)	1 (5.6)	3 (15.8)		0.97	0.614
University studies	18(14.6)	10 (13.7)	2 (11.1)	5 (26.3)		2.13	0.345
Graduate degree	79 (64.2)	48 (65.8)	14 (77.8)	10 (52.6)		2.57	0.277
PhD	3 (2.4)	2 (2.7)	1 (5.6)	0 (0)		1.07	0.587
Age, parent, *M* (*SD*)	42.67 (4.72)	42.62 (4.52)	43.22 (5.04)	41.95 (5.94)	0.32		0.728

*R-ICBT* Remitters of Internet-delivered Cognitive Behaviour Therapy; *NR-CBT* Non-remitters of Internet-delivered Cognitive Behaviour Therapy receiving additional face-to-face cognitive behaviour therapy; *NR-Decline* Non-remitters of Internet-delivered Cognitive Behaviour Therapy declining the offer to receive additional face-to-face cognitive behaviour therapy; *CAMHS* child and adolescent mental health services; *SEP* separation anxiety disorder; *GAD* generalized anxiety disorder; *SAD* social anxiety disorder; *SP* specific phobia; *PD* panic disorder; *CSR* Clinician Severity Rating derived from the Anxiety Disorder Interview Schedule

Parental anxiety and depression symptoms measured with the Hospital Anxiety and Depression Scale; Depressive symptoms self-reported with the depressive symptoms subscale of The Revised Children’s Anxiety and Depression Scale

### Long-term outcomes for remitters after ICBT

ICBT-treated remitters (*n* = 73) continued to improve on the primary outcome measure (CSR of the principal anxiety disorder), with a large effect size from post-treatment to 3MFU (*d* = 0.88; 95% CI = 0.62–1.15) and a further small effect size from 3 to 12MFU (*d* = 0.42; 95% CI = 0.17–0.68). Considering the entire trial period, from pre-treatment to 12MFU, remitters improved with a large effect size (*d* = 2.42; 95% CI = 1.78, 3.07); see Table 2 for a full summary of primary and secondary outcomes from pre-treatment to 12MFU in this group.

**Table 2:** Primary- and secondary outcome measures for remitters of ICBT (*n* = 73)

	Observed values, per protocol			Estimated change, intent-to-treat		
	Time	*n*	*M* (*SD*)	Time	Cohen’s *d* (95% CI)	*p* value
CSR	Pre	73	4.41 (0.85)	**Pre-12MFU**	2.42 (1.78, 3.07)	< 0.001
	Post	73	2.93 (0.89)	Pre–Post	1.71 (1.27, 2.15)	< 0.001
	3MFU	73	2.15 (0.88)	Post-3MFU	0.88 (0.62, 1.15)	< 0.001
	12MFU	70	1.73 (1.08)	3MFU-12MFU	0.42 (0.17, 0.68)	< 0.001
CGAS	Pre	73	58.88 (6.79)	**Pre-12MFU**	1.07 (0.76, 1.37)	< 0.001
	Post	72	66.71 (8.52)	Pre–Post	0.99 (0.74, 1.24)	< 0.001
	3MFU	70	69.67 (9.36)	Post-3MFU	0.34 (0.14, 0.55)	0.001
	12MFU	69	70.58 (10.75)	3MFU-12MFU	0.08 (− 0.09, 0.25)	0.376
RCADS-C	Pre	71	30.06 (15.48)	**Pre-12MFU**	1.14 (0.77, 1.52)	< 0.001
	Post	68	22.16 (13.83)	Pre–Post	0.54 (0.33, 0.75)	< 0.001
	3MFU	61	18.80 (12.13)	Post-3MFU	0.28 (0.07, 0.49)	< 0.001
	12MFU	52	14.62 (11.85)	3MFU-12MFU	0.34 (0.10, 0.58)	0.001
RCADS-P	Pre	73	33.37 (12.08)	**Pre-12MFU**	1.22 (0.86, 1.58)	< 0.001
	Post	73	22.44 (11.61)	Pre–Post	0.98 (0.74, 1.22)	< 0.001
	3MFU	66	20.89 (12.28)	Post-3MFU	0.11 (− 0.11, 0.33)	0.192
	12MFU	59	17.31 (11.88)	3MFU-12MFU	0.28 (0.06, 0.49)	0.003
WSAS-C	Pre	71	11.18 (7.23)	**Pre-12MFU**	1.07 (0.67, 1.48)	< 0.001
	Post	68	7.56 (6.10)	Pre–Post	0.55 (0.28, 0.82)	< 0.001
	3MFU	61	6.23 (5.63)	Post-3MFU	0.30 (0.06, 0.53)	0.019
	12MFU	52	4.12 (5.38)	3MFU-12MFU	0.34 (0.06, 0.61)	0.024
WSAS-P	Pre	73	15.82 (7.46)	**Pre-12MFU**	1.45 (1.03, 1.87)	< 0.001
	Post	73	9.95 (7.33)	Pre–Post	0.79 (0.52, 1.07)	< 0.001
	3MFU	66	7.55 (5.41)	Post-3MFU	0.33 (0.14, 0.53)	0.002
	12MFU	59	5.15 (4.94)	3MFU-12MFU	0.42 (0.16, 0.68)	0.005
KIDSCREEN-C	Pre	70	40.36 (4.65)	**Pre-12MFU**	0.41 (0.11, 0.71)	0.004
	Post	68	40.34 (5.10)	Pre–Post	0.00 (− 0.21, 0.21)	0.990
	3MFU	61	40.44 (5.69)	Post-3MFU	0.01 (− 0.18, 0.21)	0.894
	12MFU	52	42.63 (4.78)	3MFU-12MFU	0.34 (0.04, 0.65)	0.003
KIDSCREEN-P	Pre	73	37.22 (3.77)	**Pre-12MFU**	0.48 (0.21, 0.75)	< 0.001
	Post	73	38.60 (4.21)	Pre–Post	0.34 (0.14, 0.54)	0.008
	3MFU	66	38.55 (5.17)	Post-3MFU	0.02 (− 0.25, 0.22)	0.863
	12MFU	59	36.69 (4.94)	3MFU-12MFU	0.22 (− 0.01, 0.45)	0.034

*ICBT* internet-delivered cognitive behavioural therapy; *CSR* Clinician Severity Rating; *CGAS* Children’s Global Assessment Scale; *RCADS-C* or *P* Revised Children’s Anxiety and Depression Scale–child and parent versions; *WSAS-C or P* Work and Social Adjustment Scale–Child and parent versions; KIDSCREEN-C or P = KIDSCREEN-10-child and parent versions. *Note.* Mean and standard deviation based on the observed data; effect size (Cohen’s *d*) and *p* value based on the estimated means derived from the linear mixed model; RCADS-C/P Anxiety symptoms sub-scale only; the missing data due to either (1) drop-out, (2) parent and/or child forgetting to, or not wanting to, log in on platform to answer questionnaires or (3) Assessor forgetting to log assessment in case report form

Seven participants (10.3%) relapsed from being in remission at 3MFU to meeting criteria for their principal anxiety disorder at 12MFU. Thus, a total of 61 participants (89.7%) were still in remission at 12MFU. Further, 53 participants (77.9%) were free from all anxiety disorders at 12MFU.

Seven participants (10.3%) in the R-ICBT group received additional treatment elsewhere (i.e., local CAMHS) during the follow-up period: three participants who were on SSRI medication prior to starting ICBT increased their dosage during the follow-up period and four participants were referred to their local CAMHS by a clinician in the trial during the follow-up period. The results remained largely unchanged when the receipt of additional treatments during the follow-up were introduced in the models as a covariate (*p* = 0.871 for the additional treatment by time interaction effect).

### Long-term outcomes for non-remitters after ICBT

Thirty-seven participants were assessed as non-remitters at 3MFU. Eighteen of the non-remitters (48.6%) accepted the offer to receive additional F2F CBT, of which all completed the treatment. The mean number of face-to-face sessions was *M* = 6.72 (*SD* = 2.59; min–max = 2–10); supplementary Table 6 lists the perceived reasons why ICBT had not worked for these participants.

Despite not achieving remission status at post-treatment, these patients still improved significantly from pre- to post-ICBT (*t* = 3.95, *p* = 0.001; *d* = 0.99; 95% CI = 0.30–1.67). However, they improved the most after receiving additional F2F CBT (i.e., between 3 and 6MFU; *t* = 4.62, *p* < 0.001) with a large effect size (*d* = 1.53; 95% CI = 0.55–2.51). No change in the symptom severity was observed between post-treatment and 3MFU (*t* = 0.77, *p* = 0.446). Considering the entire treatment received (ICBT plus F2F CBT), the overall effect size from pre-ICBT to 12MFU on the CSR was very large (*d* = 2.27; 95% CI = 1.03–3.50); see Supplementary Table 7 for a full summary of primary- and secondary outcomes from pre-treatment to 12MFU for participants in this group.

At 6MFU, directly after having completed F2F CBT, 12 participants (70.6%) no longer fulfilled their principal anxiety disorder and ten participants (58.8%) were free from all their anxiety disorders. At 12MFU, 14 participants (82.4%) were in remission and ten participants (58.8%) were free from all their anxiety disorders. No participants relapsed between 6 and 12MFU.

Nineteen non-remitters after ICBT turned down the offer to receive additional treatment at 3MFU, of whom 36.8% (*n* = 7) did not want to and did not receive any additional treatment during the entire follow-up. Twelve participants (63.2%) turned down the offer to receive additional treatment due to already receiving additional treatment at their local CAMHS. All these participants had been referred to their local CAMHS by a clinician working in the trial. Four participants were referred due to being in need of additional treatment for an anxiety disorder and not being able to wait until 3MFU when additional treatment would be offered as part of the trial. The other eight participants were referred due to another mental health condition other than their principal anxiety disorder. For a full description and overview of referrals to local CAMHS for non-remitters turning down the offer to receive additional F2F CBT due to receiving treatment elsewhere, see Supplemental Fig 2.

Participants in NR-Decline did not improve significantly from pre- to post-treatment (*t* = 1.47, *p* = 0.148) or between post-treatment and 3MFU (*t* = 0.78, *p* = 0.442). However, they did improve significantly between 3 and 12MFU (*t* = 5.84, *p* < 0.001) with a large effect size (*d* = 1.12; 95% CI = 0.48–1.76). Considering the whole study period, the overall effect size from pre-ICBT to 12MFU on the CSR was large (*d* = 1.51; 95% CI = 0.69–2.34); see Supplementary Table 8 for a full summary of primary and secondary outcomes from pre-treatment to 12MFU for participants in this group.

**Fig. 2:**
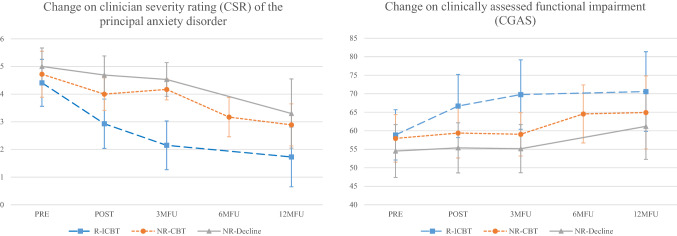
Change on primary outcome measure, clinician severity rating of the principal anxiety disorder, CSR, (left) as well as clinically assessed functional impairment, CGAS (right) based on whether participants three-months after completed ICBT were in remission (R-ICBT; *n* = 73), not in remission receiving additional face-to-face CBT (NR-CBT; *n* = 18), or not in remission declining the offer to receive additional face-to-face CBT (NR-Decline; *n* = 19). Assessments points in relation to when ICBT was completed; Six-months follow-up (6MFU) only for participants receiving additional face-to-face CBT, i.e., corresponds to the post face-to-face CBT assessment

**Fig. 3:**
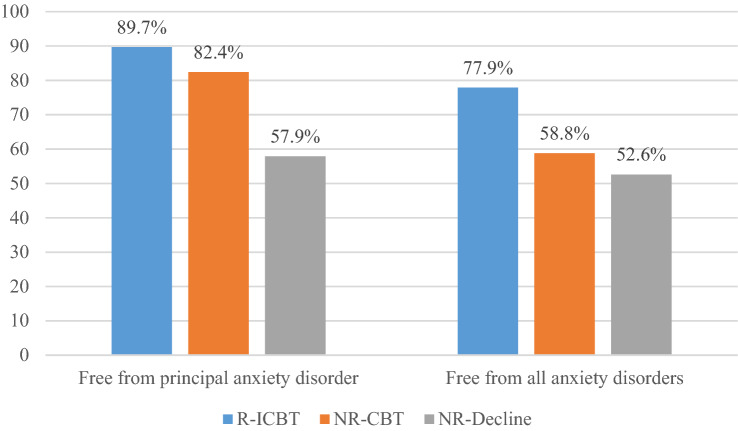
Proportion of participants in remission at 12MFU. *R-ICBT* participants assessed as remitters at three-months after completed ICBT; *NR-CBT* participants assessed as not in remission receiving additional face-to-face CBT 3-months after completed ICBT; *NR-Decline* participants not in remission declining the offer to receive additional face-to-face CBT 3-months after completed ICBT

Figure 2 shows the change over time for participants in the R-ICBT, NR-CBT and NR-Decline groups on clinician severity rating (CSR) of the principal anxiety disorder as well as clinician-assessed functional impairment (CGAS). At 12MFU, eleven participants (57.9%) in NR-Decline were assessed as being in remission and ten participants (52.6%) were free from all anxiety disorders. Figure 3 shows proportions of participants in the R-ICBT, NR-CBT and NR-Decline free from their principal anxiety disorder as well as free from all their anxiety disorders at 12MFU. No severe adverse events were reported from either ICBT- or F2F CBT treatment.

## Discussion

Whether the effects of ICBT for paediatric anxiety disorders are durable is an important clinical question. The current trial is the largest to evaluate the long-term outcomes of ICBT for paediatric anxiety disorders and the first to carefully document and statistically control for supplementary service use during the follow-up period. In addition, this study is the first to adopt a stepped-care approach in a clinical setting, whereby non-remitters to ICBT were systematically offered manualised F2F CBT; thus, providing unique insights into the feasibility of implementing this approach in regular healthcare.

The results clearly showed that treatment gains from ICBT were not only maintained, but even further improved at 12-months follow-up. This was true for clinician-, parent and self-rated anxiety symptoms and functional impairment. Few remitters relapsed (10.3%) and a clear majority were in remission (89.7%) at 12MFU. Drop-out and the missing data were low (2.9%) for the primary outcome measure CSR and clinical pre-treatment variables (such as severity of the disorder) did not differ significantly for those having missing data as compared to those providing data, suggesting that the findings were robust. The long-term effects seen in this study are on a par with and somewhat better than, previous trials of internet-delivered CBT as well as traditional face-to-face CBT for paediatric anxiety disorder [9, 10, 27]. Few participants sought additional treatment elsewhere during the follow-up period and statistically adjusting for this additional service use did not modify the results.

Non-remitters to ICBT who received additional F2F CBT (NR-CBT) improved significantly with large effects on the primary outcome measure as well as on clinician-rated functional impairment (CGAS). The largest improvement occurred after F2F CBT. By the end of the 12MFU, these patients had improved as much as those who initially responded to ICBT. These results are in line with two other trials naturalistically investigating the effects of additional CBT for children with anxiety disorders who did not respond sufficiently after first receiving parental-guided CBT [15] or attention bias modification training [14].

Somewhat unexpectedly, only 18 participants (48.6%), out of the 37 non-remitters, accepted the offer of additional F2F CBT at 3MFU. Participants declining the offer to receive additional treatment were more severe at pre-treatment, had a higher number of comorbid anxiety disorders and higher rates of participants with social anxiety disorder. Though many of these patients received treatments elsewhere, participants declining additional F2F CBT did not improve as much as participants in the other two groups. As this is a naturalistic study, we are cautious not to attribute these differences to the receipt of additional F2F CBT. Most of the participants declining the offer to receive additional treatment at 3MFU, did so because they had already sought additional treatment elsewhere. This could indicate that, for at least a sub-group of individuals, stepping up treatment should be considered earlier than 3MFU. There is a clear need to conduct further research into the identification of early predictors of treatment response in ICBT to better guide clinical decision making. Intuitively, ICBT may not be the first treatment of choice for patients with severe anxiety disorders or with complex comorbidities. However, this may not necessarily be the case; some participants who had higher level of anxiety and comorbidity at pre-treatment, responded well to ICBT and additional F2F CBT and maintained their gains at follow-up.

Strengths of this study include the large sample size, high participant retention and limited missing data. Additional strengths were the systematic recording of, and statistical adjustment for, additional treatments during the follow-up and the implementation of highly structured F2F manual in the context of a stepped-care approach. This study also had some limitations. First, this was a naturalistic follow-up study and, as such, we could not control for the passage of time. This is unfortunately the case for the majority of trials investigating the long-term outcomes of psychological treatments for paediatric anxiety disorders [28]. Thus, some of the observed improvements may be due to other factors such as environmental changes or normal maturation of the children. Second, participants in this study were less severe with regards to the severity of their principal anxiety disorder compared to other trials on ICBT for childhood anxiety disorders (e.g., [8].). Thus, the results may not generalise to the most severe end of the anxiety disorder spectrum. Third, we did not randomise participants into a stepped-care approach or a “gold standard” treatment, which would be the ideal design to test, e.g., hypothesised health economic benefits of a stepped care approach. Notwithstanding, we believe that our study highlights the feasibility of implementing such a model in regular healthcare and encourages further work in this area.

## Conclusion

This study reported on the long-term naturalistic outcomes of participants in a clinical trial of ICBT for paediatric anxiety disorders within a stepped-care model where non-remitters were offered additional face-to-face (F2F) treatment. Patients classified as remitters after ICBT maintained their gains and even improved further 1 year after the end of treatment, even when statistically controlling for supplementary service use throughout the follow-up period. Non-remitters after ICBT who choose to receive additional F2F CBT improved similarly as remitters of ICBT, the largest improvement occurring after F2F CBT. It may be feasible to implement ICBT in regular healthcare adopting a stepped care approach.

## Electronic supplementary material

Below is the link to the electronic supplementary material.

Supplementary file1 (DOCX 127 kb)
