# Supplementary material for: Long-term outcomes of internet-delivered cognitive behaviour therapy for paediatric anxiety disorders: towards a stepped care model of health care delivery
Source: Eur Child Adolesc Psychiatry. 2020 Sep 22;30(11):1723–32. doi: 10.1007/s00787-020-01645-x (PMC8558186; doi:10.1007/s00787-020-01645-x)
Supplement: Supplementary file 1 — Supplementary file1 (DOCX 127 kb) [file 787_2020_1645_MOESM1_ESM.docx]

*Supplementary material for:* **Long-term Outcomes of Internet-delivered Cognitive Behaviour Therapy for Paediatric Anxiety Disorders: Towards a Stepped Care Model of Health Care Delivery**

Maral Jolstedt^1 2^ MSc, Sarah Vigerland^1 2^ PhD, David Mataix-Cols^1 2^ PhD, Brjánn Ljótsson^3^ PhD, Tove Wahlund^1 2^ MSc, Martina Nord^1 2^ MSc, Jens Högström^1 2^ PhD, Lars-Göran Öst^4^ PhD, Eva Serlachius^1 2^ PhD

^1^Centre for Psychiatry Research, Department of Clinical Neuroscience, Karolinska Institutet, Stockholm, Sweden; ^2^Stockholm Health Care Services, Stockholm County Council, Stockholm, Sweden; ^3^Division of Psychology, Department of Clinical Neuroscience, Karolinska Institutet, Stockholm, Sweden; ^4^Department of Psychology, Stockholm University, Stockholm, Sweden.

*Corresponding author:* Maral Jolstedt, MSc, Child and Adolescent Psychiatry Research Centre, Gävlegatan 22, 113 30 Stockholm, Sweden; e-mail: maral.jolstedt@ki.se; telephone: +0851452215.

| Index | | |
| --- | --- | --- |
| Page | Table or Figure | Heading |
| 3 | Supplementary Figure 1 | An overview of the participant flow prior to being included in the current trial. from the original randomized controlled trial by Jolstedt et al. (2018). |
| 4 | Supplementary Table 1 | Inclusion- and exclusion criteria in the original randomized controlled trial by Jolstedt et al. (2018). |
| 5 | Supplementary Table 2 | List of measures. |
| 6 | Supplementary Table 3 | Time-point for when each measure was collected. |
| 7 | Supplementary Table 4 | Treatment content of the BiP Anxiety programme. |
| 8 | Supplementary Table 5 | Content of F2F treatment based on how often, *n* (%), it was used throughout treatment. |
| 9 | Supplementary Table 6 | Reasons why ICBT had not worked according to the child- and parent receiving additional F2F CBT as part of the trial (*n*=18). |
| 10 | Supplementary Figure 2 | Time point for when and for what referrals to local CAMHS was conducted by a clinician in the trial, for non-remitters turning down the offer to receive additional F2F CBT due to receiving treatment elsewhere (*n*=12). |
| 11 | Supplementary Table 7 | Primary- and secondary outcome measures for non-remitters of ICBT receiving additional face-to-face CBT (*n*=18). |
| 12 | Supplementary Table 8 | Primary- and secondary outcome measures for non-remitters of ICBT declining the offer to receive additional face-to-face CBT (*n*=19). |


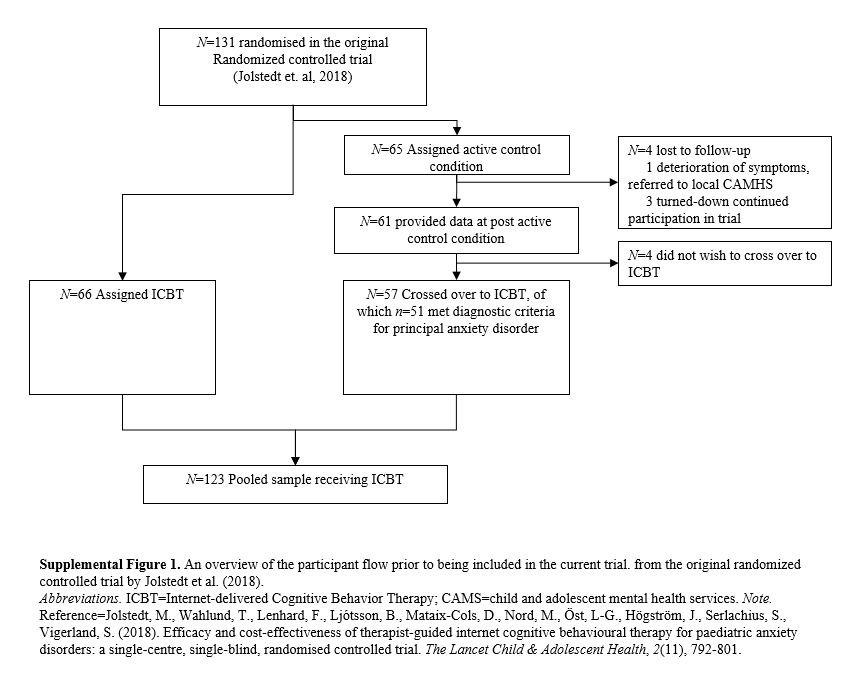


| **Supplementary Table 1.** Inclusion- and exclusion criteria in the original randomised controlled trial | |
| --- | --- |
| Inclusion criteria | |
| 1 | Being 8-12 years of age |
| 2 | Having a principal anxiety disorder of either separation anxiety disorder, social anxiety disorder, generalized anxiety disorder, panic disorder, or specific phobia |
| 3 | Having the ability to read and write in Swedish |
| 4 | Having daily access to the internet |
| 5 | Having a parent that can participate in the treatment |
| 6 | If on psychotropic medication, having a stable dose at least six weeks prior to assessment |
| Exclusion criteria | |
| 1 | Having a diagnosis of autism spectrum disorder, severe eating disorder, psychosis, or bipolar disorder. |
| 2 | Having current risk of suicide |
| 3 | Having ongoing substance dependence |
| 4 | Have received four or more sessions of exposure therapy the past six months |
| *Note.* Having a principal anxiety disorder defined as having ≥4 in the Clinician Severity Rating derived from the Anxiety Disorder Interview Schedule. | |

| **Supplementary Table 2.** List of measures. | | | |
| --- | --- | --- | --- |
| Category | Clinician assessed | Child-reported | Parent reported |
| Diagnostic status/comorbidity | ADIS |  |  |
| Anxiety symptoms severity | CSR | RCADS-C | RCADS-P |
| Impairment | CGAS | WSAS-C | WSAS-P |
| Quality of life |  | KIDSCREEN-C | KIDSCREEN-P |
| Parental anxiety- and depression |  |  | HADS |
| Experienced adverse events |  | Open-ended question | Open-ended question |
| Received additional treatment elsewhere |  | Open-ended question | Open-ended question |
| *Abbreviations.* ADIS=Anxiety Disorder Interview Schedule; CSR=Clinician Severity Rating; CGAS=Children’s Global Assessment Scale; RCADS-C or P=Revised Children’s Anxiety and Depression Scale–child and parent versions; WSAS-C or P=Work and Social Adjustment Scale–Child and parent versions; KIDSCREEN-C or P=KIDSCREEN-10–child and parent versions; HADS=Hospital Anxiety and Depression Scale. | | | |

| **Supplementary Table 3.** Time-point for when each measure was collected. | | | | | | | | |
| --- | --- | --- | --- | --- | --- | --- | --- | --- |
| Measure | Pre ICBT | Week 3 | Week 6 | Week 9 | Post ICBT | 3MFU | 6MFU | 12MFU |
| ADIS | x |  |  |  |  |  |  |  |
| CSR | x |  |  |  | x | x | x | x |
| CGAS | x |  |  |  | x | x | x | x |
| RCADS-C | x | x | x | x | x | x | x | x |
| RCADS-P | x | x | x | x | x | x | x | x |
| WSAS-C | x |  | x |  | x | x | x | x |
| WSAS-P | x |  | x |  | x | x | x | x |
| KIDSCREEN-C | x |  |  |  | x | x | x | x |
| KIDSCREEN-P | x |  |  |  | x | x | x | x |
| HADS | x |  |  |  |  |  |  |  |
| Adverse events |  |  |  |  | x |  | x |  |
| Additional treatment |  |  |  |  | x | x | x | x |
| *Abbreviations.* ICBT=Internet-delivered Cognitive Behaviour Therapy; ADIS=Anxiety Disorder Interview Schedule; CSR=Clinician Severity Rating; CGAS=Children’s Global Assessment Scale; RCADS-C or P=Revised Children’s Anxiety and Depression Scale–child and parent versions; WSAS-C or P=Work and Social Adjustment Scale–Child and parent versions; KIDSCREEN-C or P=KIDSCREEN-10–child and parent versions; HADS=Hospital Anxiety and Depression Scale. *Note.* Only participants receiving additional treatment were assessed and filled in questionnaires at 6MFU. | | | | | | | | |

| **Supplementary Table 4**. Treatment content of the BiP Anxiety programme. | | |
| --- | --- | --- |
|  | **BiP Anxiety Child program** | **BiP Anxiety Parent program** |
| Phase 1: Psychoeducation and goals | | |
| Module 1 | Psychoeducation, functional analysis | Psychoeducation, functional analysis, parental behaviours |
| Module 2 | Functional analysis, coping strategies | Functional analysis, parental behaviours, goals and hierarchies |
| Module 3 | Psychoeducation, goals and hierarchies | Functional analysis, parental behaviours, reward system, managing obstacles for exposure |
| Phase 2: Exposure | | |
| Module 4 | Psychoeducation, exposure,  reward systems; for panic disorder interceptive exposure; for social anxiety disorder social awareness training | Functional analysis, parental behaviours, exposure |
| Module 5 | Functional analysis, exposure, managing obstacles for exposure | Functional analysis, parental behaviours, exposure |
| Module 6 | Functional analysis, exposure | Exposure, problem solving |
| Module 7 | Functional analysis, exposure,  cognitive restructuring; for generalized anxiety disorder imaginal exposure | Exposure |
| Module 8 | Functional analysis, exposure,  problem solving | Exposure, keeping motivation |
| Module 9 | Functional analysis, exposure | Exposure, getting help from others |
| Module 10 | Functional analysis, exposure | Exposure |
| Module 11 | Functional analysis, exposure | Exposure |
| Phase 3: Maintenance and relapse prevention | | |
| Module 12 | Maintenance and relapse prevention | Maintenance and relapse prevention |
| *Note.* Coping strategies involves breathing techniques, relaxation skills and mindfulness. | | |

| **Supplementary Table 5.** Content of F2F treatment based on how often, *n* (%), it was used throughout treatment. | | | | | | |
| --- | --- | --- | --- | --- | --- | --- |
|  |  | | | | | |
|  | Total | SEP | SAD | SP | GAD | PD |
| Participants, *n* | 18 | 5 | 5 | 4 | 3 | 1 |
| Sessions (total), *n* | 121 | 28 | 44 | 22 | 18 | 9 |
| **Treatment content, *n* (%)** |  |  |  |  |  |  |
| Goals and Exposure hierarchies | 43 (35.5) | 10 (35.7) | 11 (25.0) | 6 (27.3) | 8 (44.4) | 8 (88.9) |
| Psychoeducation | 43 (35.5) | 16 (57.1) | 13 (29.5) | 6 (27.3) | 6 (33.3) | 2 (22.2) |
| Stress management /relaxation training | 11 (9.1) | 2 (7.1) | 2 (4.5) | 4 (18.2) | 2 (11.1) | 1 (11.1) |
| Functional analysis | 32 (26.4) | 12 (42.9) | 9 (20.5) | 3 (13.6) | 4 (22.2) | 4 (44.4) |
| Exposure in vivo | 49 (40.5) | 3 (10.7) | 24 (54.5) | 10 (45.5) | 11 (61.1) | 1 (11.1) |
| Exposure as homework assignment | 105 (86.8) | 26 (92.9) | 38 (86.4) | 18 (81.8) | 14 (77.8) | 9 (100) |
| Parental behaviors | 31 (25.6) | 9 (32.1) | 5 (11.4) | 5 (22.7) | 10 (55.6) | 2 (22.2) |
| Problem solving | 7 (5.8) | 4 (14.3) | 0 (0) | 1 (4.5) | 1 (5.6) | 1 (11.1) |
| Reward systems | 21 (17.4) | 13 (46.4) | 1 (2.3) | 1 (4.5) | 6 (33.3) | 0 (0) |
| Cognitive restructuring | 29 (24.0) | 12 (42.9) | 10 (22.7) | 3 (12.6) | 2 (11.1) | 2 (22.2) |
| *Abbreviations.* CBT=cognitive behavioral therapy SEP=separation anxiety disorder; GAD=generalized anxiety disorder; SAD=social anxiety disorder; SP=specific phobia; PD=panic disorder. | | | | | | |

| **Supplementary Table 6.** Reasons why ICBT had not worked according to the child- and parent receiving additional F2F CBT as part of the trial (*n*=18). | |
| --- | --- |
| Child had not done any exposure tasks or had not done enough exposure tasks, *n* (%) | 10 (55.6) |
| Child had worked actively with exposure during ICBT and had experienced a decrease in symptoms, but still fulfilled diagnostic criteria, *n* (%) | 5 (27.8) |
| Child improved during ICBT but relapsed between post treatment and 3MFU, *n* (%) | 3 (16.7) |
| *Abbreviations.* ICBT=Internet-delivered cognitive behaviour therapy. | |


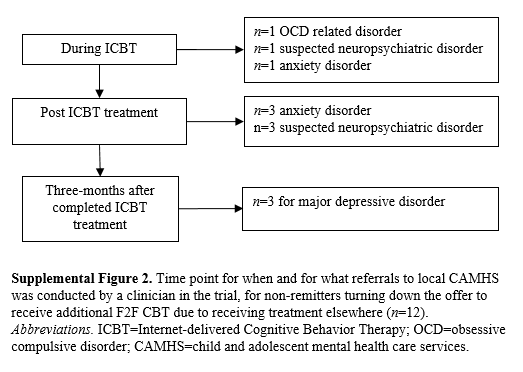


| **Supplementary** **Table 7.** Primary- and secondary outcome measures for non-remitters of ICBT receiving additional face-to-face CBT (*n*=18). | | | | | | | | | |
| --- | --- | --- | --- | --- | --- | --- | --- | --- | --- |
|  | Observed values, per protocol | | | | Estimated change, intent-to-treat | | | | |
|  | Time | | *n* | *M* (*SD*) | Time | Cohen’s *d* (95% CI) | | *p*-value | |
| CSR | Pre  Post  3MFU  6MFU  12MFU | | 18  18  18  18  18 | 4.72 (0.83)  4.00 (0.59)  4.17 (0.38)  3.17 (0.71)  2.89 (0.76) | **Pre-12MFU**  Pre-Post  Post-3MFU  3MFU-6MFU  6MFU-12MFU | 2.27 (1.03, 3.50)  0.99 (0.30, 1.67)  -0.33 (-0.96, 0.29)  1.53 (0.55, 2.51)  0.38 (-0.09, 0.85) | | <0.001  0.001  0.446  <0.001  0.205 | |
| CGAS | Pre  Post  3MFU  6MFU  12MFU | | 18  18  18  18  18 | 57.94 (6.42)  59.39 (6.73)  59.06 (5.87)  64.56 (7.84)  64.17 (9.87) | **Pre-12MFU**  Pre-Post  Post-3MFU  3MFU-6MFU  6MFU-12MFU | 0.85 (-0.35, 2.04)  0.22 (-0.14, 0.58)  -0.05 (-0.31, 0.21)  0.89 (0.49, 1.29)  0.04 (-0.37, 0.46) | | 0.002  0.274  0.795  0.002  0.762 | |
| RCADS-C | Pre  Post  3MFU  6MFU  12MFU | | 18  16  17  13  14 | 31.67 (17.40)  30.75 (14.42)  26.65 (15.73)  19.77 (8.60)  18.86 (8.32) | **Pre-12MFU**  Pre-Post  Post-3MFU  3MFU-6MFU  6MFU-12MFU | 0.80 (0.16, 1.44)  0.11 (-0.13, 0.36)  0.12 (-0.03, 0.28)  0.36 (0.03, 0.68)  0.12 (-0.33, 0.57) | | 0.001  0.379  0.243  0.031  0.620 | |
| RCADS-P | Pre  Post  3MFU  6MFU  12MFU | | 18  17  17  11  15 | 33.06 (15.22)  27.18 (13.78)  24.47 (13.23)  24.36 (10.03)  20.53 (10.06) | **Pre-12MFU**  Pre-Post  Post-3MFU  3MFU-6MFU  6MFU-12MFU | 0.68 (0.10, 1.27)  0.43 (0.21, 0.65)  0.11 (-0.20, 0.42)  0.09 (-0.23, 0.41)  0.08 (-0.33, 0.49) | | 0.001  <0.001  0.338  0.563  0.706 | |
| WSAS-C | Pre  Post  3MFU  6MFU  12MFU | | 18  16  17  13  14 | 10.61 (7.95)  10.50 (7.30)  8.18 (7.29)  9.23 (6.38)  5.43 (5.19) | **Pre-12MFU**  Pre-Post  Post-3MFU  3MFU-6MFU  6MFU-12MFU | 0.60 (0.06, 1.13)  0.06 (-0.29, 0.41)  0.28 (-0.02, 0.58)  -0.08 (-0.27, 0.10)  0.47 (0.15, 0.79) | | 0.021  0.709  0.076  0.619  0.030 | |
| WSAS-P | Pre  Post  3MFU  6MFU  12MFU | | 18  17  17  11  15 | 13.00 (8.64)  11.76 (7.31)  12.47 (7.58)  10.09 (6.58)  8.00 (6.40) | **Pre-12MFU**  Pre-Post  Post-3MFU  3MFU-6MFU  6MFU-12MFU | 0.53 (-0.13, 1.19)  0.20 (-0.09, 0.48)  -0.13 (-0.44, 0.17)  0.23 (-0.24, 0.71)  0.30 (-0.14, 0.74) | | 0.036  0.179  0.361  0.298  0.161 | |
| KIDSCREEN-C | Pre  Post  3MFU  6MFU  12MFU | | 18  16  17  13  14 | 40.28 (4.66)  40.13 (3.01)  39.82 (3.83)  40.31 (4.33)  38.14 (5.16) | **Pre-12MFU**  Pre-Post  Post-3MFU  3MFU-6MFU  6MFU-12MFU | -0.39 (-1.19, 0.42)  0.04 (-0.44, 0.51)  -0.21 (-0.66, 0.24)  0.09 (-0.46, 0.64)  -0.36 (-1.11, 0.36) | | 0.130  0.879  0.479  0.750  0.154 | |
| KIDSCREEN-P | Pre  Post  3MFU  6MFU  12MFU | | 18  17  17  11  15 | 37.61 (4.29)  37.06 (4.63)  38.00 (4.87)  38.09 (2.47)  37.40 (4.22) | **Pre-12MFU**  Pre-Post  Post-3MFU  3MFU-6MFU  6MFU-12MFU | -0.05 (-1.05, 0.94)  -0.08 (-0.49, 0.33)  0.15 (-0.23, 0.53)  0.13 (-0.23, 0.50)  -0.41 (-1.61, 0.79) | | 0.750  0.705  0.456  0.581  0.140 | |
| *Abbreviations.* ICBT=internet-delivered cognitive behavioural therapy; F2F CBT=face-to-face cognitive behaviour therapy; CSR=Clinician Severity Rating; CGAS=Children’s Global Assessment Scale; RCADS-C or P=Revised Children’s Anxiety and Depression Scale–child and parent versions; WSAS-C or P=Work and Social Adjustment Scale–Child and parent versions; KIDSCREEN-C or P=KIDSCREEN-10–child and parent versions. *Note.* Three-months follow-up assessment (3MFU) was the pre face-to-face CBT assessment and six-months follow-up assessment was the post face-to-face assessment; Mean and standard deviation based on observed data; Effect size (Cohen’s *d*) and *p*-value based on estimated means derived from the linear mixed model; RCADS-C/P Anxiety symptoms sub-scale only; Missing data due to either (1) drop-out, (2) parent and/or child forgetting to, or not wanting to, log in on platform to answer questionnaires, or (3) Assessor forgetting to log assessment in case-report form. | | | | | | | | | |
| **Supplementary Table 8.** Primary- and secondary outcome measures for non-remitters of ICBT declining the offer to receive additional face-to-face CBT (*n*=19). | | | | | | | | |  |
|  | | Observed values, per protocol | | | Estimated change, intent-to-treat | | | |  |
|  | | Time | *n* | *M* (*SD*) | Time | Cohen’s *d* (95% CI) | *p*-value | |  |
| CSR | | Pre  Post  3MFU  12MFU | 19  17  19  17 | 5.00 (0.68)  4.71 (0.69)  4.53 (0.61)  3.24 (1.25) | **Pre-12MFU**  Pre-Post  Post-3MFU  3MFU-12MFU | 1.51 (0.69, 2.34)  0.46 (0.00, 0.91)  0.24 (-0.17, 0.66)  1.12 (0.48, 1.76) | <0.001  0.148  0.442  <0.001 | |  |
| CGAS | | Pre  Post  3MFU  12MFU | 19  17  19  16 | 54.53 (7.14)  55.71 (6.77)  55.16 (6.51)  62.38 (8.91) | **Pre-12MFU**  Pre-Post  Post-3MFU  3MFU-12MFU | 0.78 (0.10, 1.46)  0.12 (-0.44, 0.68)  -0.03 (-0.33, 0.26)  0.73 (0.28, 1.17) | 0.008  0.630  0.876  <0.001 | |  |
| RCADS-C | | Pre  Post  3MFU  12MFU | 18  12  12  6 | 31.39 (14.69)  27.33 (11.91)  23.92 (9.01)  25.50 (7.34) | **Pre-12MFU**  Pre-Post  Post-3MFU  3MFU-12MFU | 1.26 (-0.52, 3.04)  0.44 (-0.14, 1.03)  0.31 (-0.10, 0.71)  -0.04 (-1.44, 1.35) | 0.038  0.037  0.125  0.916 | |  |
| RCADS-P | | Pre  Post  3MFU  12MFU | 18  16  15  10 | 40.72 (8.73)  34.31 (8.45)  32.47 (7.41)  28.50 (10.52) | **Pre-12MFU**  Pre-Post  Post-3MFU  3MFU-12MFU | 1.42 (0.31, 2.53)  0.83 (0.16, 1.51)  0.21 (-0.52, 0.95)  0.49 (-0.34, 1.32) | <0.001  0.001  0.434  0.097 | |  |
| WSAS-C | | Pre  Post  3MFU  12MFU | 18  12  12  5 | 13.56 (6.78)  9.83 (4.51)  7.08 (4.36)  10.00 (4.95) | **Pre-12MFU**  Pre-Post  Post-3MFU  3MFU-12MFU | 1.10 (-0.08, 2.28)  0.62 (-0.09, 1.34)  0.39 (-0.25, 1.03)  0.40 (-1.10, 1.90) | <0.001  <0.001  0.019  0.024 | |  |
| WSAS-P | | Pre  Post  3MFU  12MFU | 18  16  15  10 | 18.61 (6.97)  15.13 (8.54)  14.47 (8.22)  12.00 (6.70) | **Pre-12MFU**  Pre-Post  Post-3MFU  3MFU-12MFU | 1.37 (0.16, 2.59)  0.75 (0.13, 1.37)  0.27 (-0.38, 0.91)  0.29 (-0.49, 1.06) | <0.001  <0.001  0.002  0.005 | |  |
| KIDSCREEN-C | | Pre  Post  3MFU  12MFU | 18  12  12  5 | 37.94 (4.43)  37.75 (4.75)  37.33 (6.96)  37.60 (5.77) | **Pre-12MFU**  Pre-Post  Post-3MFU  3MFU-12MFU | 0.03 (-1.29, 1.35)  -0.28 (-0.88, 0.33)  0.10 (-0.14, 0.34)  0.23 (-0.82, 1.27) | 0.947  0.328  0.457  0.340 | |  |
| KIDSCREEN-P | | Pre  Post  3MFU  12MFU | 18  16  15  10 | 33.89 (4.31)  35.44 (4.10)  33.93 (4.51)  34.60 (4.55) | **Pre-12MFU**  Pre-Post  Post-3MFU  3MFU-12MFU | 0.17 (-1.03, 0.70)  0.39 (-0.29, 1.07)  -0.32 (-0.86, 0.22)  0.21 (-0.49, 0.91) | 0.624  0.194  0.256  0.488 | |  |
| *Abbreviations.* ICBT=internet-delivered cognitive behavioural therapy; CSR=Clinician Severity Rating; CGAS=Children’s Global Assessment Scale; RCADS-C or P=Revised Children’s Anxiety and Depression Scale–child and parent versions; WSAS-Y or P=Work and Social Adjustment Scale–Youth and parent versions; KIDSCREEN-C or P=KIDSCREEN-10–child and parent versions.  *Note.* Mean and standard deviation based on observed data; Effect size (Cohen’s *d*) and *p*-value based on estimated means derived from the linear mixed model; RCADS-C/P Anxiety symptoms sub-scale only; Missing data due to either (1) drop-out, (2) parent and/or child forgetting to, or not wanting to, log in on platform to answer questionnaires, or (3) Assessor forgetting to log assessment in case-report form. | | | | | | | | |  |
